# Simulating temperature distribution of high-intensity focused ultrasound during bone treatments

**DOI:** 10.1186/2050-5736-3-S1-P6

**Published:** 2015-06-30

**Authors:** Thomas Hudson, Thomas Looi, Adam Waspe, James Drake, Samuel Pichardo

**Affiliations:** 1 Hospital for Sick Children, Toronto, Canada; 2 Centre for Image Guided Innovation and Therapeutic Intervention, Toronto, Canada; 3 Thunder Bay Research Institute/Lakehead University, Toronto, Canada

## Background/introduction

To help improve therapeutic results for treatment of bone tumors, high intensity focused ultrasound (HIFU) is being investigated as a non-invasive alternative to surgery and radiotherapy. HIFU ablative treatments require an imaging modality for guidance, and magnetic resonance (MR) imaging is currently the strongest choice as it is able to provide temperature mapping throughout the treatment process. Unfortunately, the temperature feedback that is critical to ensuring safety and optimal soft tissue ablation is often unobtainable in bone structures. As an alternative to obtaining feedback, this project is focused on developing a simulator that is capable of predicting the temperature distribution in and around bone tissue during HIFU therapy. Having such a simulation would provide a better understanding of the thermal dosages incurred by the bone during HIFU treatment of tumors.

## Methods

The simulator consists of three primary components: semi-automatic segmentation, acoustic field simulation and the bio-heat transfer model. The segmentation is performed using a mesh-based method, applying Gaussian statistics to conform to the outer surface. The acoustic field uses a modified solution to the Rayleigh integral to track the change in complex ultrasonic velocity potential through solid and soft tissue. The velocity potentials at each voxel are then converted to form a volumetric heat distribution required by the bio-heat model. This model then computes a numerical solution to the heat transfer problem, creating a four dimensional temperature map capable of demonstrating specific time intervals throughout a therapeutic ultrasound procedure. The simulation has been implemented using parallel GPU architecture, capable of producing large 4D bone maps in under one minute.

## Results and conclusions

**Figure 1:**
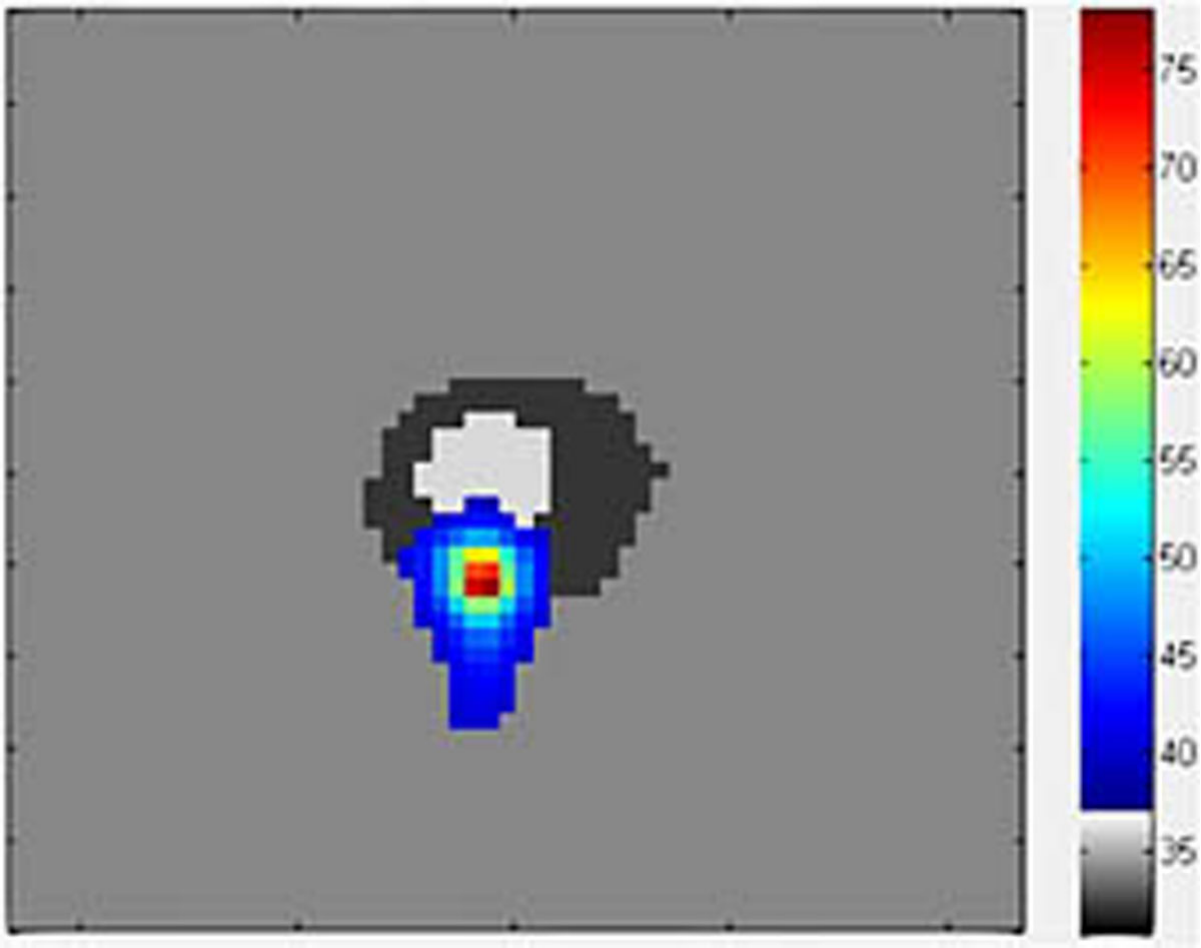
Simulated therapy on segmented bone, 40W applied for 30 seconds. Target is close to center of bone.

**Figure 2:**
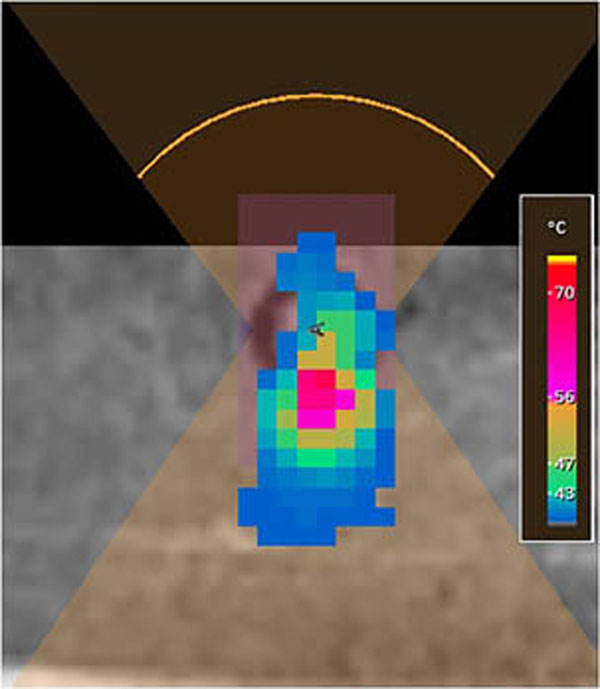
Porcine experiment with the same bone, target and parameters as in previous figure.

Figure 1 demonstrates the final bio-heat model from a 40W sonication of 30s duration. The heat appears to accumulate mostly in the cortical layer, reaching 77.2°C at its peak cortical temperature. Repeated acoustic simulations required between 18 and 22 seconds of computation time and bio-heat between 6 and 9 seconds. Previous MR thermometry data (Figure 2) of an experiment on a porcine femur yielded a maximum temperature of 73.4°C during a 40W, 20s treatment, almost four degrees lower than the simulation. Heat also appeared to spread more freely into the bone during the actual therapy. Differences in material properties are likely to account for a large part of these discrepancies, as many reported porcine properties are highly variable across the literature.

The next steps of this project will be to conduct a material property study of both healthy and cancerous tissue, and to collect enough data to characterize the accuracy and validate the functionality of the model. With a fully validated prototype, this software tool will be able to start demonstrating the thermal effects of HIFU in various bone structures, and will help both physicians and scientists in understanding the full effects of this therapy for improved HIFU procedures.

